# Thyrotoxic Periodic Paralysis as an Ongoing Diagnostic Challenge: A Case Report and Literature Review

**DOI:** 10.7759/cureus.46272

**Published:** 2023-09-30

**Authors:** Shourya Tadisina, Reda Asad, Aishwarya Varakantam, Lamont Weide, Betty Drees

**Affiliations:** 1 Endocrinology, Diabetes and Metabolism, University of Missouri Kansas City School of Medicine, Kansas City, USA; 2 College of Medicine, Boston University, Boston, USA

**Keywords:** diagnostic challenge, thyrotoxic hypokalemic periodic paralysis, missed diagnosis, delayed diagnosis, thyrotoxic periodic paralysis

## Abstract

Thyrotoxic periodic paralysis (TPP) is a rare condition that presents with episodic periodic paralysis due to hypokalemia that develops from hyperthyroidism. Timely diagnosis is still an ongoing challenge due to lack of awareness, self-resolving episodes, and the fact that it clinically mimics familial hypokalemic periodic paralysis (FHPP), which is more common in the West. TPP is more commonly seen among Asians but has been emerging in Western countries due to globalization. We present a case of a 24-year-old Hispanic male who presented with bilateral lower extremity weakness. He had five such episodes in the past year, which resolved on their own. The current episode of weakness was worse, and he required a wheelchair to ambulate. Despite extensive work, it took over four months to make a definitive diagnosis and treat his hyperthyroidism. A literature review reported that most cases of TPP are usually diagnosed after multiple episodes, and the causes of diagnostic error were studied. Through this review, we present a case of TPP with diagnostic delay, a literature review discussing the etiology, pathogenesis, clinical manifestations, and management, with an emphasis on the diagnostic challenge of TPP. Awareness of this condition, timely evaluation for hyperthyroidism as a cause for hypokalemic periodic paralysis, and understanding the factors that contribute to its diagnostic challenge will aid in timely recognition and treatment.

## Introduction

An association between thyrotoxicosis and periodic paralysis was established in 1902 by Rosenfeld [1]. Thyrotoxic periodic paralysis (TPP) is most often reported in Asians, but its incidence has been growing in Western countries due to globalization. It is commonly misdiagnosed as familial hypokalemic periodic paralysis in the West, which is an autosomal dominant condition, while TPP is sporadic. About 2% of patients with thyrotoxicosis in China and Japan have TPP, while the incidence of TPP among non-Asians in the United States is only one tenth (0.1%-0.2%) of what is seen in Asian countries [2]. Several studies have shown a higher incidence in males than females, with a ratio of 20:1, despite the higher incidence of hyperthyroidism in females [2]. Genetic predisposition in Asians is thought to be associated with the human leucocyte antigen DRw8 isotype (HLA-DRw8), but the exact mechanism is still not understood [3].

Interestingly, we encountered two patients with TPP at our practice recently. One was diagnosed early and treated accurately due to the typical clinical presentation, while the other patient had delayed diagnosis and treatment. We present here the case of delayed diagnosis, review the pathophysiology, presentation, and treatment of TPP, and discuss the diagnostic challenges of TPP based on reported cases.

## Case presentation

A 24-year-old Hispanic male with no past medical history presented with episodic weakness of bilateral lower extremities with spontaneous recovery in a couple of days. He had five episodes in the year before presentation, with worsening weakness requiring wheelchair assistance during the last few episodes. He was seen by a primary care provider for the bilateral lower extremity weakness, and lab work revealed a potassium level of 2.7 mmol/L (normal range 3.6 to 5.2 mmol/L), thyroid stimulating hormone (TSH) of <0.05 mIU/L (normal 0.5-5 mIU/L), and free thyroxine (FT4) of 7.77 ng/dl (normal 0.9 to 2.3 ng/dl). He was started on potassium replacement 20 milliequivalents daily and referred to neurology and endocrinology for further evaluation.

Neurologic evaluation revealed bilateral lower extremity tremulousness, hyperreflexia in all extremities, and mild proximal muscle weakness of the bilateral lower extremities. There was fragmentation of information available, and the neurologist was not aware of the associated hypokalemia and hyperthyroidism. Due to concerns about a primary neurologic pathology, the patient was admitted to the hospital for a neurologic evaluation. He was tachycardiac with a pulse of 116 bpm, and the rest of his vitals were stable. Apart from the neurologic exam described above, no other physical findings were noted. Laboratory evaluation revealed a low potassium level of 3.2 mmol/L, and the remainder of the electrolytes, kidney function, and hepatic function tests were within normal limits. Thyroid function tests were not ordered. He was given potassium replacement with some improvement in weakness. Lumbar puncture and magnetic resonance imaging (MRI) of the brain and spine ruled out any acute neurological pathology. On review of the images on the MRI by the neurologist, a thoracic arterio-venous fistula was suspected as a cause of transient paresthesia, but no definitive diagnosis was made. The patient was discharged home with outpatient follow-up. Additional diagnostic evaluations at multiple medical centers were performed over the following four months, but again without a clear diagnosis.

About four months after the initial presentation, he was evaluated by endocrinology for hyperthyroidism. This delay occurred while the neurologic workup was completed. The initial neurologic presentation with hyperreflexia was atypical for TPP, and as noted above, the neurologist did not have information about the underlying hyperthyroidism. At the time of the endocrinology evaluation, the vital signs were: temperature 98 degrees F, pulse 106 beats per minute, blood pressure 129/72 mm Hg, respiratory rate 18 breaths per minute, oxygen saturation 98% on room air, and body mass index (BMI) 32.2 kg/m2. On review of systems, he reported a 100-pound unintentional weight loss in nine months, tremors, and episodic lower extremity weakness. He was using a cane to ambulate. He denied any anxiety, palpitations, or diaphoresis. A physical exam revealed sinus tachycardia, fine tremors of the bilateral upper extremities, hyperreflexia with deep tendon reflexes +3 noted on all four extremities, motor strength 4/5, and intact sensation. He had no thyromegaly or thyroid bruit.

Pending repeat laboratory results, a presumptive diagnosis of TPP was made based on hypokalemia, hyperthyroidism, and episodic muscle weakness. He was started on propranolol 20 mg three times a day and continued potassium chloride 20 milliequivalents daily.

**Figure 1:**
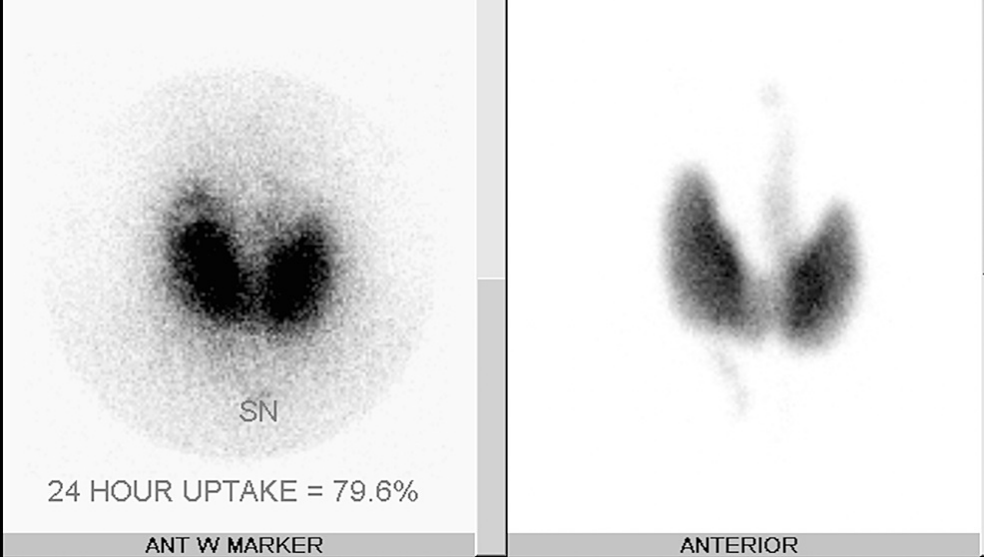
Nuclear medicine thyroid uptake and scan with technetium 99m after 13.8 mCi of iodine-131 (I-131) showed 79.6% homogenous uptake, consistent with Graves’ disease

The repeat laboratory evaluation revealed a suppressed TSH of <0.01 mIU/L (normal range: 5mIU/L) an elevated FT4 of 4.95 ng/dl (normal range: 0.9 - 2.3 ng/dl) a free triiodothyronine (FT3) of 17.28 pg/ml (normal range: 2.3 pg/ml) elevated thyroid simulating immunoglobulin (TSI) of 190 IU/L (normal <140 IU/L) and elevated thyrotrophin receptor antibodies (TRAB) of 7.24 IU/L (normal <2 IU/L). Nuclear medicine thyroid uptake and scan with technetium 99m after 13.8 mCi of iodine-131 (I-131) showed homogenous increased uptake in tracer activity throughout the thyroid gland, consistent with Graves' disease (Figure 1). He was treated with radioactive iodine ablation at 25 mCi of I-131. At follow-up a few weeks later, he was found to be hypothyroid and started on levothyroxine. He is now euthyroid on replacement therapy and has had no recurrence of TPP episodes.

## Discussion

TPP can occur from any cause of thyrotoxicosis, and the most common cause is Graves’ disease. Other etiologies include toxic nodular goiter, solitary toxic nodule, exogenous thyroxine use, amiodarone-induced thyrotoxicosis, and thyrotropin-secreting pituitary adenoma [2-4]. Differences in the genetic subtypes could be the reason for the increased prevalence in the Asian population. HLA-DRw8 in Japanese individuals and HLA-A2, Bw22, Aw-19, and B-17 in Singapore and Chinese individuals have been reported to predispose them to TPP. Genetic mutations that control the sodium-potassium ATPase [Na-K ATPase] activity within the same HLA subtype could also contribute to ethnic differences [2]. Mutations of the potassium channels Kir2.6 and other channel mutations have been shown to predispose patients to TPP [4,5]. It is usually precipitated after a heavy carbohydrate meal, during rest after strenuous activity, with high sodium intake, and with trauma [2,3]. Hyperinsulinemia is also found to be a contributing factor in precipitating TPP, as it has been reported in people with obesity [6].

The pathophysiology behind hypokalemia and periodic paralysis was not clearly understood for decades. The degree of hypokalemia correlates with the severity of muscle weakness. A complete recovery of weakness is seen at normal potassium levels. Skeletal muscle is the largest reserve of total body potassium and plays a major role in extracellular potassium homeostasis. This is done by Na-K-ATPase and potassium channels-inward rectifying and delayed rectifying channels-that regulate inward and outward potassium movement, respectively [7]. Thyroid hormone stimulates Na-K-ATPase activity by both genomic-mediated increased transcription of the Na-K ATPase pump and nongenomic-mediated increased intrinsic pump activity. Beta-2 adrenergic activity is also known to stimulate the pump [2,7].

Hyperinsulinemia seen in TPP can also stimulate the Na-K-ATPase pump and cause hypokalemia, which explains the role of a heavy-carbohydrate diet in triggering the episodes [3,4,7]. Elevated androgen levels have also been shown to increase pump activity, which is consistent with the higher incidence of TPP in males despite the higher incidence of hyperthyroidism in females [2,7].

**Figure 2:**
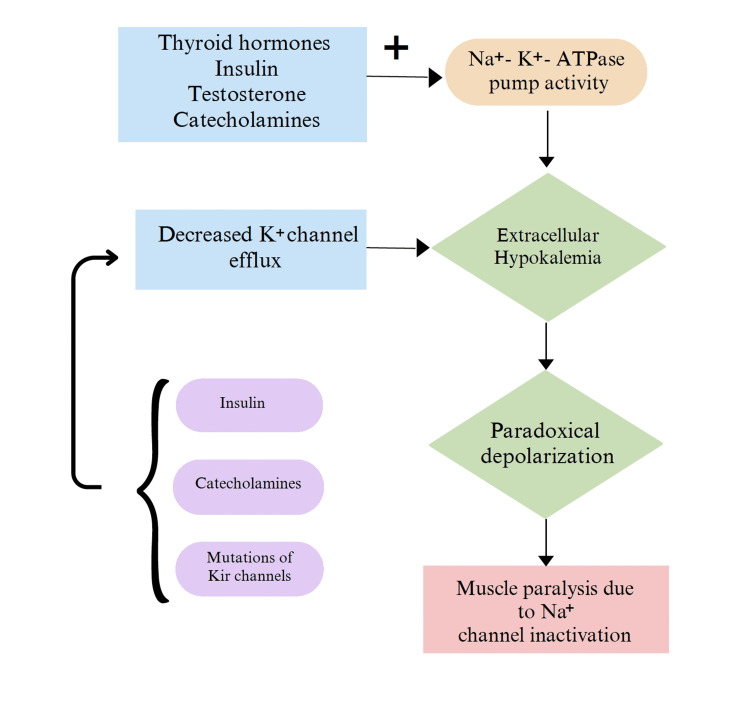
Pathophysiology of TPP Image credits: Aishwarya Varakantam, Shourya Tadisina Na+-K+-ATPase activity is stimulated by thyroid hormones, insulin, catecholamines, and testosterone. Mutations of Kir channels, insulin, and catecholamines can decrease potassium efflux, leading to an increase in intracellular potassium. The decreased K+ efflux and increased activity of the Na+-K+-ATPase pump lead to extracellular hypokalemia, paradoxical depolarization, which causes inactivation of Na+ channels, and muscle paralysis.

Loss of function mutations in the genes encoding potassium efflux channels (Kir 2.6) can prevent potassium efflux from affecting potassium homeostasis. These mutations have been described in a third of TPP patients. Insulin and catecholamines can also inhibit Kir channels. With extracellular hypokalemia, the skeletal muscle membrane develops paradoxical depolarization rather than hyperpolarization, which inactivates sodium channels, resulting in weakness and paralysis [5,7]. Figure 2 depicts the pathophysiology described above.

Initial episodes usually present between 20 and 40 years of age [4,6-9]. Presents with episodic muscle weakness that mainly affects the proximal muscles of the lower extremities; these episodes can progress to flaccid quadriplegia. Muscle weakness usually resolves spontaneously in a few hours to two days, even without potassium replacement, but hypokalemia may cause life-threatening cardiac arrhythmias and respiratory failure. The bulbar, respiratory, and ocular muscles are usually spared, but in rare situations, severe respiratory distress requiring ventilatory support and ICU admission has been reported [2-4,6,8,9].

Patients may have symptoms of hyperthyroidism, such as weight loss, tachycardia, palpitations, heat intolerance, and diaphoresis, but they are most often subtle in presentation. A physical exam is consistent with signs of thyrotoxicosis, including tachycardia, tremors, goiter, and thyroid bruit. Though thyrotoxicosis usually presents with hyperreflexia, TPP usually presents with absent or diminished reflexes [2-4,6,8,9].

Laboratory findings usually reveal hypokalemia during an acute episode. However, patients may have hypomagnesemia and hypophosphatemia, which are also due to the catecholamine-driven intracellular shifts. As hypokalemia in these patients is due to intracellular shift, there is no renal or gastrointestinal loss of potassium. Urine potassium/creatinine ratio and trans-tubular potassium gradient [urine/plasma potassium]/(urine/plasma osmolality] are usually low due to low urinary excretion of potassium [2-4,10]. Abnormal thyroid function tests with low TSH and elevated FT4 and FT3 levels are seen. Nuclear medicine thyroid uptake and scans will reveal increased, diffuse uptake in the thyroid gland. Electrocardiography reveals sinus tachycardia. The presence of U waves may be seen due to hypokalemia, prolonged PR intervals, and widened QRS complexes. Atrioventricular blocks, atrial fibrillation, and ventricular fibrillation may be present on rare occasions [2-4,10].

Emergent management to prevent cardiopulmonary complications involves careful potassium replacement. The hypokalemia is from intracellular potassium shift rather than potassium loss. Thus, normalizing the potassium levels can treat muscle paralysis but is associated with the risk of developing rebound hyperkalemia [2-4,6,8,9]. To prevent this, no more than 60 milliequivalents should be given in 24 hours [3,4]. Nonselective beta blockers, such as propranolol, may be given orally at a high dose of 3-4 mg/kg, either with potassium chloride or by itself, to abort acute episodes [7]. Recurrent attacks can be prevented by avoiding precipitating factors until a euthyroid state is achieved and by using nonselective beta blockers, such as propranolol [2-4,6,9]. There is no evidence supporting the use of potassium supplements to prevent attacks [2,3,5].

Definitive therapy is to treat the cause of thyrotoxicosis or hyperthyroidism with either antithyroid drugs, radioiodine ablation, thyroidectomy, or reducing or stopping exogenous thyroid hormone administration. Treating hyperthyroidism leads to complete resolution, making TPP a treatable and curable condition [2-4,6,8,9].

TPP is an ongoing diagnostic challenge. Reports in the literature demonstrate cases where the diagnosis was made after patients experienced multiple events of episodic paralysis. The reasons contributing to diagnostic error can be attributed to patient, provider, disease, and system-based factors. Table 1 summarizes the reasons that lead to diagnostic challenges based on our review.

**Table 1:** Displays the different factors affecting the diagnostic challenge of TPP

Factors	
Patient	Delayed presentation:due to subtle symptoms, self-resolving episodes.Gender: more common in males but can present in females as well. Ethnicity: common in Asians but reported in other different ethnicities. Family History: familial hypokalemic periodic paralysis (FHPP)
Provider	Lack of awareness of TPP as a cause of hypokalemic periodic paralysis.Focused history and physical exam:given time constraints in an acute setting.Availability bias: as FHPP is more common in the West.Anchoring bias:diagnosing other more common causes of hypokalemic periodic paralysis based on initial available information.
Disease	Self-resolving episodesclinically mimic FHPP.Atypical presentation: can present with normokalaemia and periodic paralysis, atypical neurologic symptoms.
System-based	Difficulty accessing patient records from different systems. Miscommunication among providers. Fragmentation of data.

Patient-based factors include epidemiology related to gender and ethnicity. Though Asians have a higher genetic predisposition to this condition, it has been reported in other ethnicities as well. Similarly, though more common in males, it has also been reported in females. There may be a delay in recognition for non-Asian populations and women. Since the symptoms are periodic, with resolution between episodes of paralysis, patients may delay seeking medical care early during TPP.

The disease-based factors include the fact that TPP can have mild symptoms, an atypical presentation, and may mimic FHPP. Cases with subtle symptoms of thyrotoxicosis and self-resolving episodes of paralysis seem to delay seeking care until patients experience severe debilitating episodes [8,11]. Two cases of TPP were reported with normokalaemia initially but developed delayed hypokalemia during hospitalization. Both of these cases had a known diagnosis of Graves’ disease, and one was already on treatment [12,13].

The provider-based factors include a focused history and physical exams that may miss subtle symptoms of thyrotoxicosis and a lack of awareness of TPP as a cause of hypokalemic periodic paralysis. These factors may lead to both availability bias and anchoring bias, both of which lead to delays or misdiagnosis. Availability bias is a tendency to make quick decisions based on the available information. TPP is sporadic in occurrence and commonly misdiagnosed as familial hypokalemic periodic paralysis (FHPP), which is more common in the West, or sporadic hypokalemic periodic paralysis. Diagnosis may thus be delayed until the patient has recurrent episodes of paralysis [14-16]. Anchoring bias is when a diagnosis is made based on the initial information, and there is an inability to adjust this diagnosis when further information is available. This bias can cause inadequate treatment, causing recurrent episodes before the actual diagnosis is made [17]. For example, in one of the cases, periodic paralysis was initially attributed to hypokalemia from hydrochlorothiazide use, and subsequent evaluation during the same hospitalization revealed hyperthyroidism, leading to the early diagnosis of TPP [18]. In this case, the providers were not subject to anchoring bias, and a complete workup for hypokalemic periodic paralysis was done, and an early diagnosis of TPP was made.

Multiple system-based factors may play a role in the delayed or missed diagnosis of TPP. Since thyroid function tests may be altered in acute illnesses unrelated to any underlying thyroid pathology, they are not routinely obtained in inpatient settings unless there are clinical signs of thyroid disease [19]. Thus, thyroid function tests would not generally be part of an inpatient admission. Additionally, miscommunication among providers and lack of access to medical records lead to fragmentation of clinical data, which makes it challenging for providers to make an accurate diagnosis.

Table 2 provides an overview of eight cases with delayed diagnosis reported in the literature and presents the different diagnostic challenges. The challenges are spread across patient, provider, disease, and system-based factors. A thorough history and physical exam, communication among providers, and awareness of TPP can lead to timely evaluation of thyrotoxicosis in patients with periodic paralysis and hypokalemia, but early, accurate diagnosis remains a challenge.

**Table 2:** The most common factors causing diagnostic errors in TPP based on cases reported in the literature. M: male, ED: emergency department, PtR: patient-related, PrR: provider-related, DR: disease-related, SB: system-based

Case Reports	Age in years/Sex	Ethnicity/ Race	ED visits/ number of episodes	Time to diagnosis	Potassium (K+) at Diagnosis Milliequivalent per liter (mEq/L)	Thyrotoxic symptoms	Diagnostic error factors
Barahona et al. [8]	37/M	Caucasian	4 episodes	4 months	2.3	Tremors, Heat OIntolerance Thyromegaly	DR & PtR: ethnicity, self-resolving episodes
Al Moteri et al. [11]	28 /M	Filipino	2 episodes	5 years	1.6	Tremors, Unilateral proptosis	DR & PtR: self-resolving episodes
Chakrabarti et al. [12]	27/M	-	Several episodes	3 years	Hospitalization day 1 to 4.2 ; Potassium dropped on day 3-2.8	Thyromegaly Thyroid Eye Disease	DR: atypical presentation with normokalaemia
Valizadeh et al. [13]	32/M	Iranian	Several episodes	1 year	Hospitalization day 1 to 4.49 ; Potassium dropped on day 4-2.5	Tremors Heat Intolerance	DR: atypical presentation with normokalaemia
Garla et al. [14]	23/ M	African American	1 Ed visit several episodes	6 months	1.6	Tachycardia, Exophthalmos, Thyromegaly	PtR: delayed presentation
Catalano et al. [15]	33 /M	Italian	3 ED visits	4 weeks	2.9	Tachycardia	PrR: availability bias SB: fragmentation of information
Naqi et al. [16]	20 /M	Chinese	Several episodes		3.1	Tachycardia, Tremors, Weight loss	PtR and PrR: family history of FHPP
Lam et al. [17]	33/M	Hispanic	Several episodes	2 years	1.7	Thyromegaly Tachycardia	PtR: ethnicity, delayed presentation PrR: anchoring bias
Current case	27/M	Hispanic	5 episodes	1 year	3.1	Tremors Weight Loss	PtR: ethnicity, delayed presentation DR: atypical neurologic symptoms PrR: anchoring bias SB-Fragmentation of information

## Conclusions

Our case displays a delay in diagnosis and treatment due to a combination of factors. The patient factors were the delay in presentation due to very subtle symptoms of hyperthyroidism, self-resolving episodes of periodic paralysis, and a non-Asian ethnicity with a lower incidence of TPP. Despite the presence of hyperthyroidism and hypokalemia with periodic paralysis, TPP was not immediately recognized due to an atypical presentation of the neurologic symptoms, the mildness of the hyperthyroid symptoms, and the lack of availability of outpatient laboratory results at the time of inpatient admission. The atypical neurologic presentation, which raised concern over an urgent, acute upper motor neuron pathology, resulted in initial resources directed toward neurologic evaluation and delayed endocrinology evaluation. Once diagnosed and the underlying thyrotoxicosis treated, the patient’s symptoms completely resolved, and he remains symptom-free. Although TPP is a rare type of hypokalemic periodic paralysis, timely recognition and intervention lead to complete resolution of the condition. Increasing awareness and improving coordination of care may lead to more accurate and timely diagnosis and treatment.
